# A Revolutionary Device for Endovascular Aortic Repair of Abdominal Aortic Aneurysm: A Pilot Study

**DOI:** 10.12688/f1000research.152060.2

**Published:** 2024-11-06

**Authors:** Taofan Taofan, Iwan Dakota, Sung Gwon Kang, Suko Adiarto, Suci Indriani, Ruth Grace Aurora, Rendra Mahardika Putra, Bagas Adhimurda Marsudi, Melani Limenco Benly, Macmilliac Lam, Muhammad Rafli Iqbal, Bagus Herlambang, Yoga Yuniadi, Renan Sukmawan, Bambang Widyantoro

**Affiliations:** 1 Department of Cardiology and Vascular Medicine, Faculty of Medicine University of Indonesia, University of Indonesia Academic Hospital, National Cardiovascular Center Harapan Kita, Jakarta, Jakarta, 11420, Indonesia; 2 Department of Radiology, Chosun University, Gwangju-Si, Gwangju-Si, 61452, South Korea; 3 Research Assistant of Vascular Division, Department of Cardiology and Vascular Medicine, Faculty of Medicine University of Indonesia, National Cardiovascular Center Harapan Kita, Jakarta, Jakarta, 11420, Indonesia; 4 Department of Thorax and Cardiovascular Surgeon, Faculty of Medicine University of Indonesia, University of Indonesia Academic Hospital, National Cardiovascular Center Harapan Kita, Jakarta, Jakarta, 11420, Indonesia

**Keywords:** Endovascular Aortic Repair, Abdominal Aortic Aneurysm, Stent Graft, B-EVAR, Contralateral Gate Cannulation

## Abstract

**Background:**

Endovascular repair for abdominal aortic aneurysms (AAA) has been the gold standard since it was established in 1991. Various graft configurations have been developed to overcome the limitations of endovascular aortic repair (EVAR), including contralateral gate cannulation (CGC). We propose a new device and technique intended to simplify endovascular AAA repair, along with reports of its application in six patients.

**Intervention:**

The Taofan and Kang (T&K) bidirectional endovascular aortic repair (B-EVAR (B-EVAR) device uses a main-body stent graft, two-limb extensions, and bare metal stent. The procedure involved accessing the right and left femoral arteries, followed by standard aortography evaluation using a pigtail catheter through the contralateral femoral access. The main body stent graft is deployed through ipsilateral femoral access using a balloon catheter, while the contralateral gate is cannulated with a hydrophilic coated wire. Both balloons were inflated simultaneously to ensure that the wires were in different lumens. Contralateral and ipsilateral extensions were deployed using a limb extension stent graft. Standard aortography evaluation was repeated.

**Result:**

T&K B-EVAR pilot procedures proved successful in various cases, from simple to complex anatomy, and even with varied graft stent deployment strategies. None of the patients had complications or prolonged length of stay (LOS). Follow-up CT did not reveal any high-pressure endoleaks.

**Conclusion:**

T&K B-EVAR has been proven to simplify endovascular AAA repair in six patients with excellent results. It is also reproducible, potentially universally applicable, and can offer operators ease of use, faster procedure times, reduced fluoroscopy times, and reduced contrast usage.

## Introduction

Abdominal Aortic Aneurysm (AAA) is defined as a dilation equal to or greater than 3 cm in size or an increase of ≥1.5 times the normal diameter at the renal artery level. ^1^^,^^2^ Its prevalence increases with age. The current global prevalence of AAA in patients aged 60 years or older is estimated to be around 1.2-3.3%. ^2^ While in the Asian population, the overall prevalence was around 2.56% in patients with cardiovascular risk factors. ^3^ In 2017, it was reported that AAA caused 167,000 deaths and 3 million disability-adjusted life years worldwide. ^4^

Intervention is recommended for AAA patients with rapid aneurysm growth (>5 mm/6 months) or a fusiform aneurysm with a maximum diameter of 5.5 cm or more. Studies have shown that endovascular aortic repair (EVAR) provides better results than open repair in terms of perioperative mortality rate, 30-day mortality rate, procedure time, blood loss, and length of stay (LOS). ^5^ EVAR procedures require experienced operators in well-equipped centers with proper devices and operating rooms. They are expected to have sufficient training to perform catheter-based interventions with the recommended minimum number of endovascular procedures, including 80 endovascular therapeutic procedures, 100 endovascular diagnostic procedures, and 20 EVARs. ^6^^,^^7^ Furthermore, EVAR has been shown to use considerable contrast when performed. ^8^ Thus, various graft configurations have been developed over time to subjugate the limitations that come with EVAR, including one of the most challenging and time-consuming parts of EVAR, which is the contralateral gate cannulation (CGC). ^9^ We propose a new device and technique intended to simplify endovascular AAA repair alongside reports of its application on six patients.

## Method

The procedures were performed at the National Cardiovascular Center in Harapan Kita, Jakarta, Indonesia, which saw approximately 55 EVAR and thoracic endovascular aortic repair (TEVAR) cases per year. A vascular intervention consultant cardiologist with more than ten years of experience performed the procedures, assisted by a fellow vascular intervention student.

Prior to conducting this study, we sought ethical approval from the National Cardiovascular Center in Harapan Kita. The stages involved in obtaining ethical approval included submitting the research proposal, developing the initial study protocol, creating a prototype, conducting stent graft trials, and ultimately presenting at a full board meeting. We received ethical clearance from the National Cardiovascular Center in Harapan Kita under the reference number DP.04.03/KEP161/EC086/2023 on September 12, 2023. The ethics committee authorized the study, ensuring that the research subjects’ rights are protected and confirming that the research adheres to the ethical, legal, social, and non-clinical standards outlined in the Nuremberg Code and the Helsinki Declaration.

The Taofan & Kang Bidirectional Endovascular Aortic Repair (T&K B-EVAR) is a newly developed universal device aimed at addressing limitations observed in conventional bifurcated EVAR systems. This innovative design converts the traditional bifurcated EVAR technique into a tubular or trunk-type EVAR, using limb extensions deployed based on the Taofan-Kang Modified Mitsudo’s Kissing Balloon Formula. The B-EVAR device enhances technical feasibility by streamlining contralateral gate cannulation and reducing procedural and fluoroscopy time.

The inclusion criteria for the procedure were based on the anatomical suitability for the T&K B-EVAR device, consistent with standard bifurcated EVAR anatomical requirements. These included a proximal infra-renal aortic neck of at least 15 mm, distal iliac arteries of 10–15 mm, and a minimum external iliac artery diameter of 7 mm. The exclusion criteria mirrored those of standard EVAR, including unfavorable anatomical features such as excessive aortic tortuosity, circumferential calcification, significant thrombus burden, and access vessels of small caliber.

**Figure 1.**
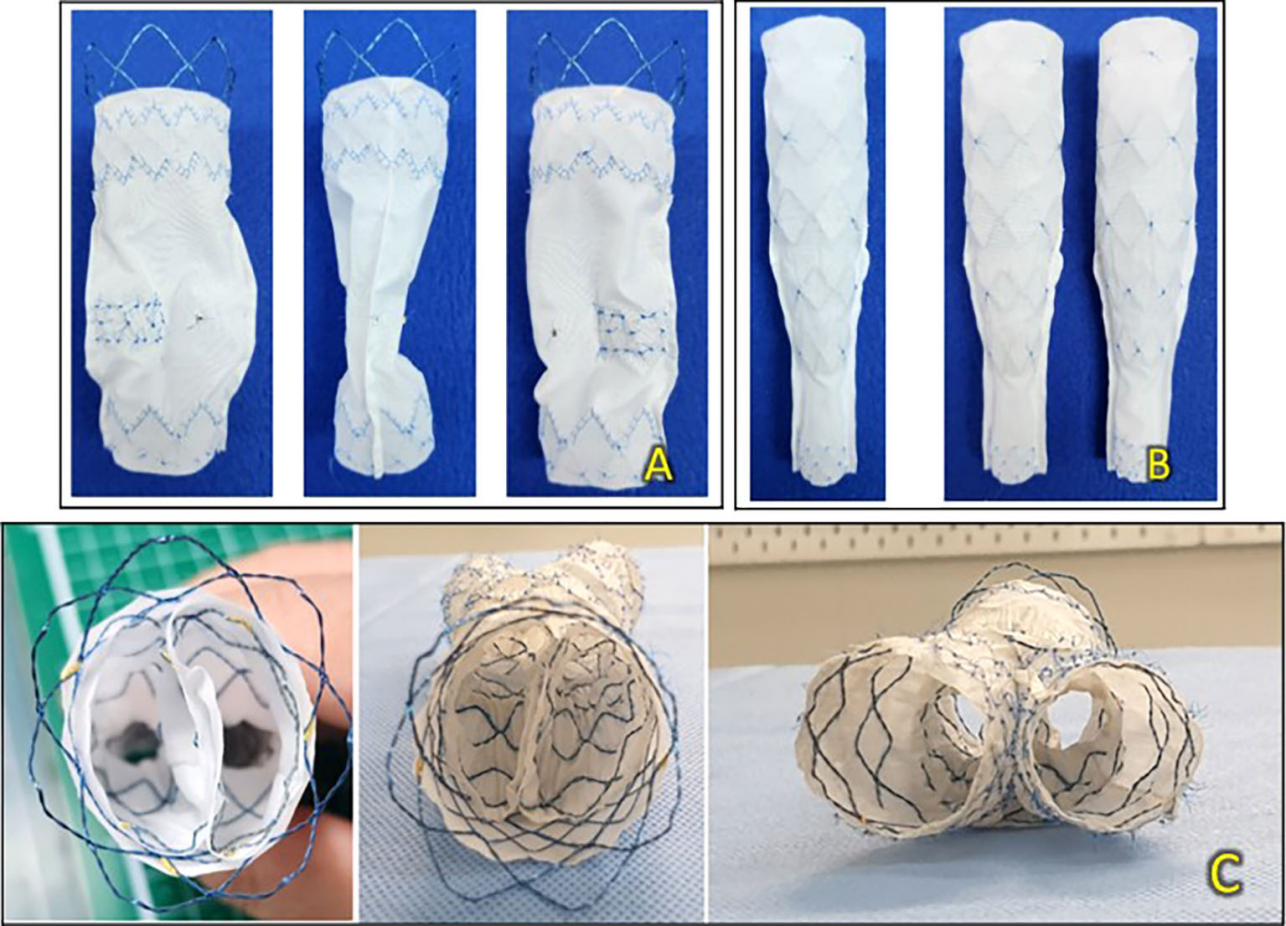
T&K B-EVAR Devices. (A) Primary body stent graft from the front, side, and back. (B) Limb extensions from the front and side. (C) Axial view of the device.

The device consists of a main body stent graft with a bare stent and encapsulated two-limb extensions. As shown in the accompanying figures, the septum within the T&K B-EVAR main body stent graft is centrally positioned but extends only to the lower middle portion of the main body, leaving the distal section free. This design provides adequate space for contralateral cannulation, enabling adjunctive procedures when necessary, thereby maintaining procedural flexibility for complex interventions ( Figure 1A-B).

### T&K B-EVAR procedure

The standard procedure requires two access sites from the bilateral common femoral arteries, which are achieved by surgical cutdown. Standard aortography was performed using a 5 Fr marker pigtail catheter through the contralateral femoral artery. The guidewires were placed in the ascending aorta. Both wires were replaced with an extra stiff wire. Subsequently, both contralateral and ipsilateral femoral artery sheaths are exchanged with larger sheath sizes ranging from 16 F to 18 F SEAL NOVUS Body Stent Graft™ (S&G Biotech, Yongin, Korea) deployment is performed through the more patent, more anatomically ideal access site. A balloon catheter is placed on the ipsilateral side. At the same time, the contralateral gate was cannulated using a 0.035 hydrophilic coated wire with the assistance of a multipurpose catheter, and another balloon catheter was placed. Both balloons were inflated simultaneously to ensure that they were in different lumens. If both balloons are successfully inflated, it can be inferred that both wires successfully cannulated the separate lumens. The contralateral and ipsilateral limb extensions were deployed using a SEAL NOVUS Limb stent graft™ (S&G Biotech, Yongin, Korea). The standard aortography evaluation procedure was performed to evaluate the graft stent position, apposition, endoleaks, and patency of the renal arteries. Many follow the same procedure in the six cases presented. However, certain cases have highlighted the applicability of T&K B-EVAR, even with challenging anatomical morphology.

## Case presentation

### Case 1

**Figure 2.**
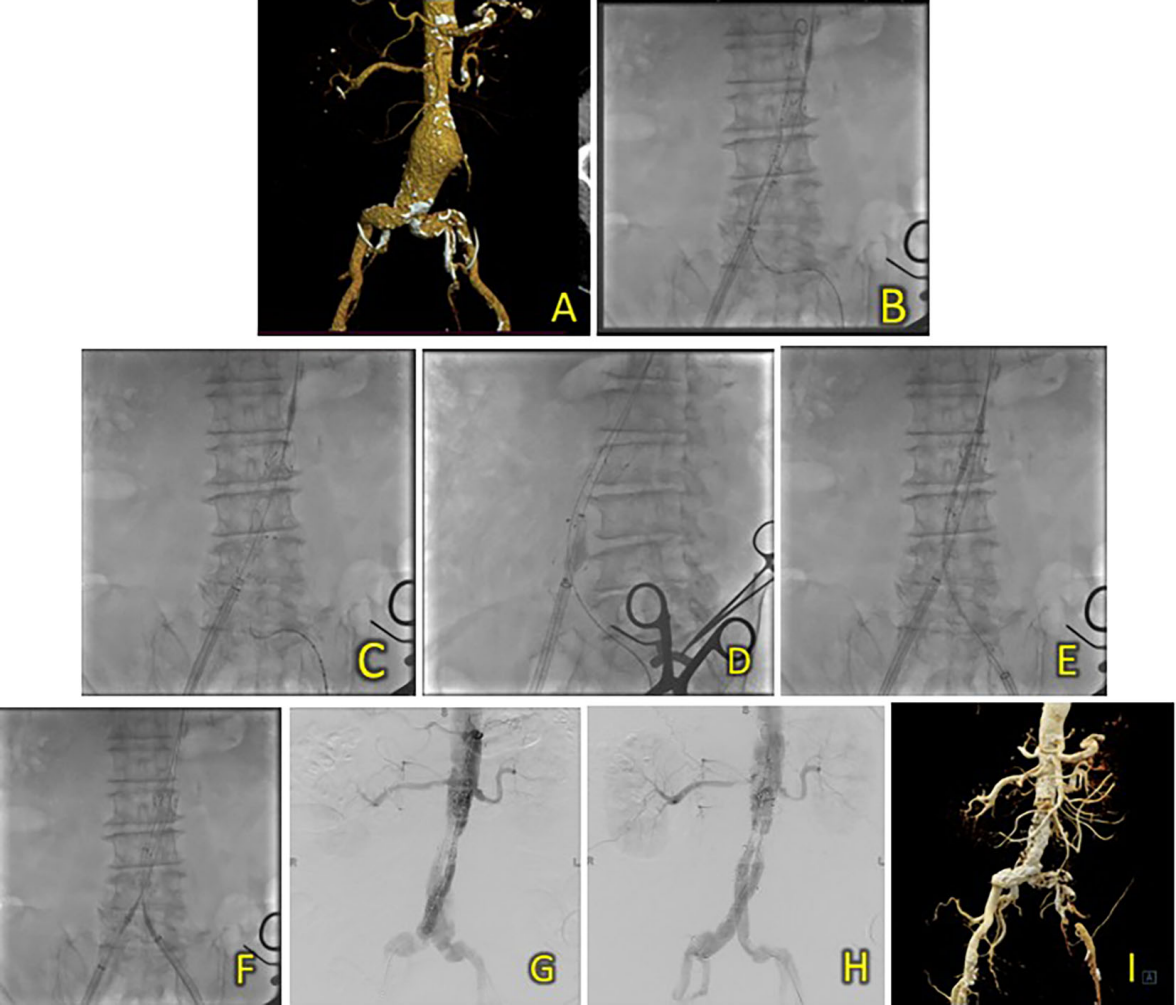
T&K B-EVAR on Patient 1. (A) CT of the 1 ^st^ patient’s aortic pathology. (B) Diagnostic angiography. (C-H) T&K B-EVAR step-by-step with limb extension deployment using a ballerina technique. (I) Post-procedure follow-up CT.

An 83-year-old man was admitted with sharp, pulsing abdominal pain 1 year prior to presentation. The patient also had a history of hypertension. Laboratory results showed normal hemoglobin counts (Hb 12.2 mg/dL) and mildly reduced eGFR (Ureum 35.6 mg/dL, Creatinine 1.33 mg/dL, and eGFR 53 ml/min/1.73 m ^2^). A CT-Scan angiography (CTA) was performed. It revealed an AAA from the infrarenal region to the aortic bifurcation 79 mm in length and a maximum sac diameter of 58.2 mm, and dilatation of the right common iliac artery with a diameter of 21.1 mm ( Figure 2A). A T&K B-EVAR procedure was then performed for the patient.

Surgical cutdown was performed to gain access to the right and left femoral arteries. Initial aortography revealed an infrarenal fusiform abdominal aorta aneurysm ( Figure 1B). A SEAL NOVUS Body Stent Graft 26(20) × 90 mm × 15 × 550 (S&G Biotech, Yongin, Korea) was deployed through the right femoral artery. Subsequent wiring was performed using a 0.035 hydrophilic coated wire, guided by an MP2/5F catheter. Deployment of the SEAL NOVUS Body Stent Graft 22(12) × 120 mm 18 × 550 mm (S&G Biotech, Yongin, Korea) was performed through ipsilateral access ( Figure 2C-G). Aortography showed the stent in a good position, a type 1 B endoleak, and stenosis in the intrastent abdominal aorta. Contralateral and ipsilateral limb extensions using the SEAL NOVUS Limb Stent Graft 12 (24) × 80 mm 15 × 550 mm (S&G Biotech, Yongin, Korea) were deployed ( Figure 2H). When withdrawing the olive tip through ipsilateral femoral access, it was difficult to retract the apparatus. Hence, we continued to snare from the brachial access site, which allowed us to free the apparatus. Aortography revealed a minimal endoleak from the right iliac artery and intrastent stenosis at the abdominal aorta. Post-dilatation of the contralateral iliac stent was performed using a balloon catheter 10-46 mm inflated to 9 cc. Contralateral and ipsilateral intrastent post-dilatation were performed simultaneously using peripheral 10 × 60 mm and 10 × 60 mm balloons, respectively. Final angiography showed good stent position and no residual endoleaks or intrastent stenosis ( Figure 2H). The total contrast was 130 mL hexiol, and the dose area product (DAP) was 205.48 Gy.cm ^2^. The total procedure time was 1 h and 50 min, and the fluoroscopy times were 41 min and 56 s, respectively.

The patient was discharged without any complaints after four days. Follow-up CTA performed 3 days and 2 months after the procedure showed a deployed stent in the infrarenal aorta until the left and right common iliac arteries without endoleaks ( Figure 2I).

### Case 2

**Figure 3.**
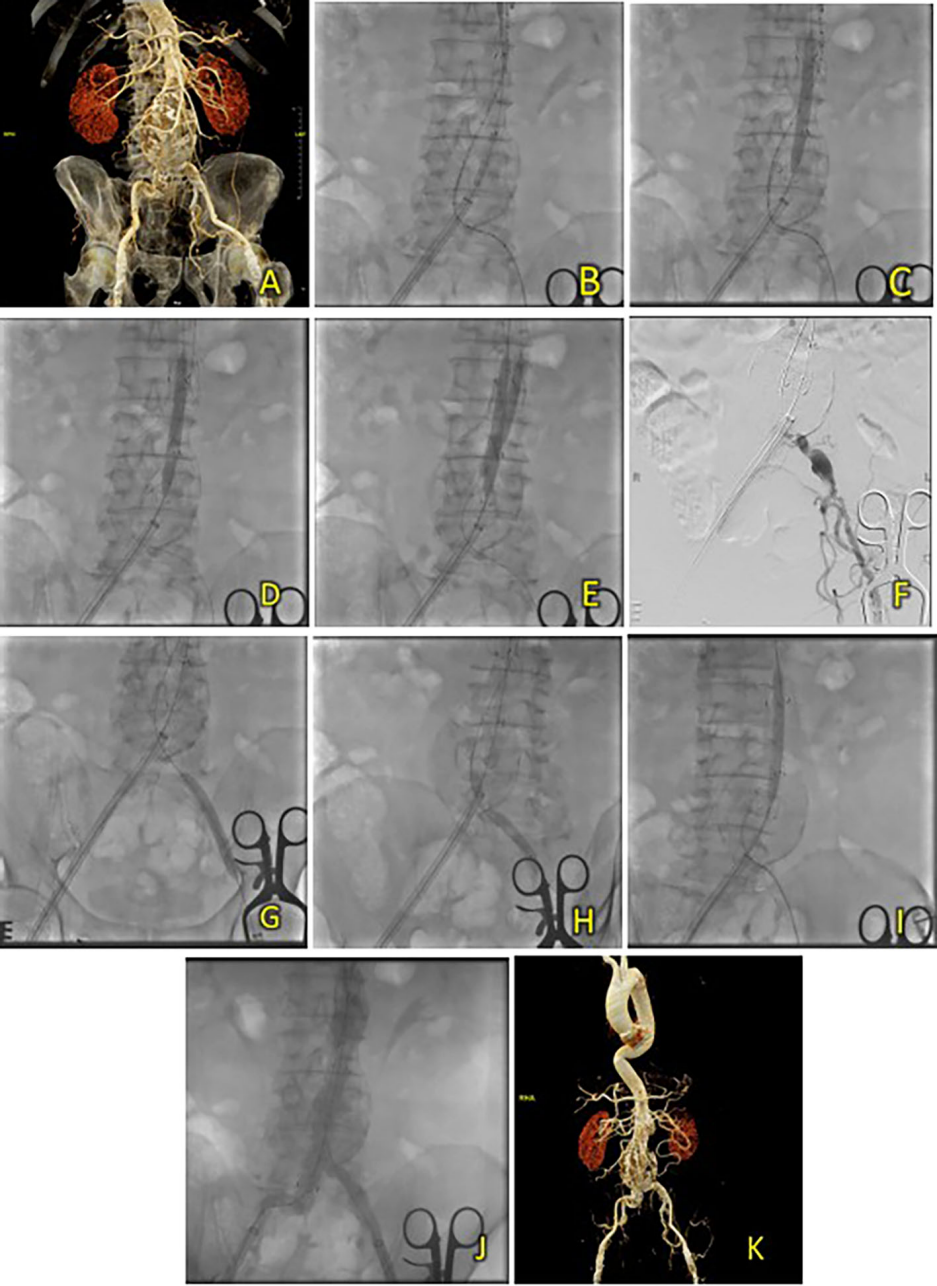
T&K B-EVAR on Patient 2. (A) Pre-procedure CT of the 2 ^nd^ patient’s aortic pathology. (B-E) Deployment of T&K B-EVAR. (F-J) Stenosis of the left iliac artery and subsequent ballooning with good results. (K) Post-procedure CT scan evaluation.

A 72-year-old man was referred with an infrarenal abdominal aortic aneurysm. The patient also had a history of hypertension, diabetes, and dyslipidemia. Lab results showed anemia (Hb 10.9 mg/dL), elevated blood urea level (49.8 mg/dL), normal creatinine (1.23 mg/dL), and normal eGFR (62 ml/min/1.73 m ^2^). CT-Scan Angiography (CTA) revealed abdominal aortic aneurysm with thrombus and irregular calcification from the infrarenal region below the bilateral renal arteries until aortic bifurcation around 121.4 mm in length and a maximum sac diameter of 71.2 mm, with thrombosed and dilated right common iliac artery with a diameter of 20.4 mm ( Figure 3A). A T&K B-EVAR procedure was then scheduled.

A standard T&K B-EVAR procedure was performed. After femoral artery access, a SEAL NOVUS Body Stent Graft was deployed, followed by limb extensions. Balloon dilation resolved stenosis in the left iliac artery. Final aortography confirmed good stent position with no endoleak, and renal arteries remained patent ( Figure 3B-J). Total contrast used was 70 mL of Iopamiro 370, with a dose area product (DAP) of 201.02 Gy.cm ^2^. The total procedure time was 1 hour 10 minutes, and fluoroscopy time was 33 minutes 37 seconds.

After five days, the patient was discharged without any complaints. Follow-up CTA within the same month showed good graft position and no endoleak ( Figure 3K).

### Case 3

**Figure 4.**
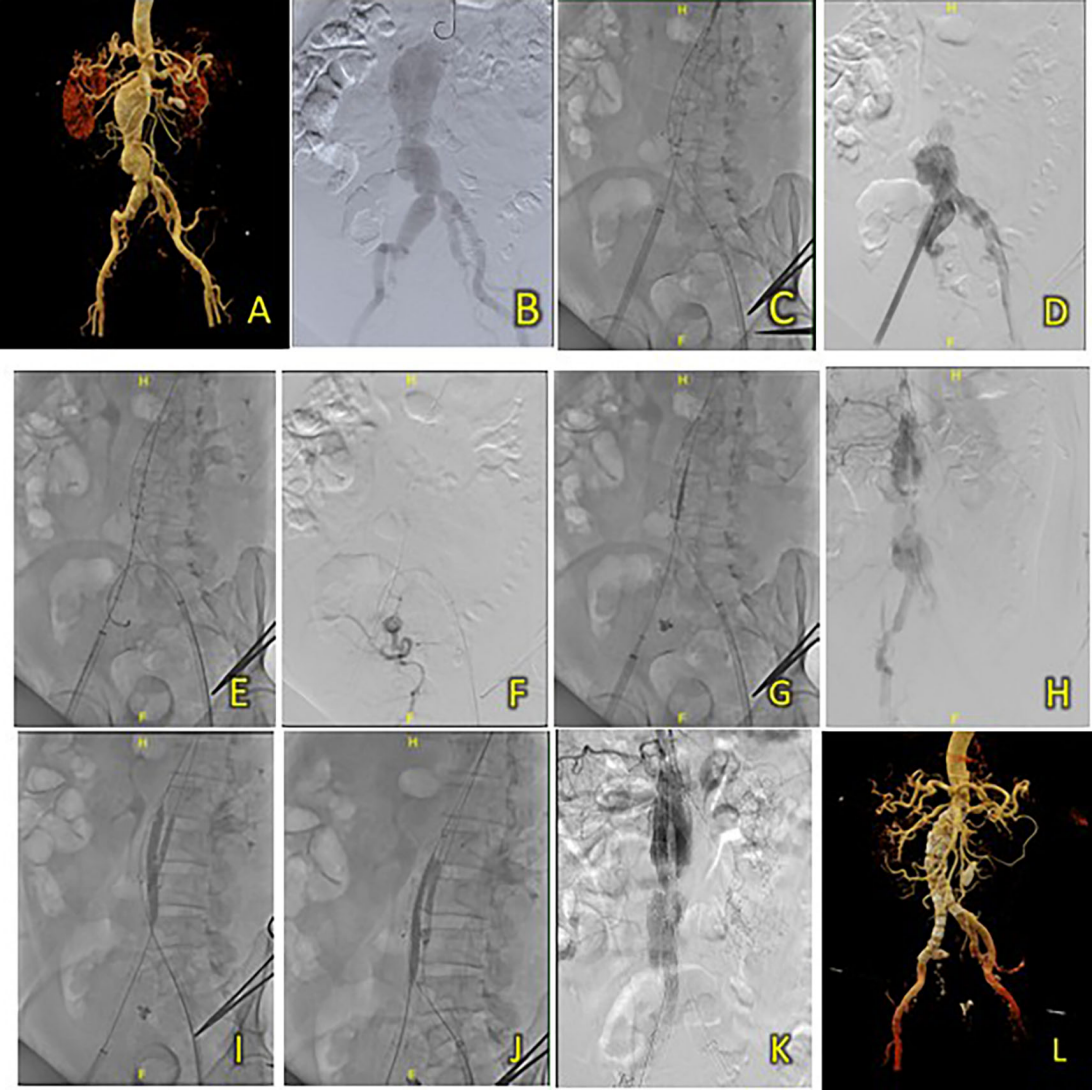
T&K B-EVAR on Patient 3. (A) Pre-procedure CT scan of the 3 ^rd^ patient’s aortic pathology. (B) Diagnostic angiography. (C-D) Standard deployment of T&K B-EVAR with angiography evaluation. (E-F) Coiling of right internal iliac artery and coronary angiography evaluation. (G-H) The deployment of limb extensions and evaluation showed slow flow through the graph stent. (I-J) Balloon dilatation intrastent. (K) Evaluation angiography without endoleaks. (L) Post-procedure CT scan evaluation.

A 67-year-old male was initially suspected of having an abdominal aortic aneurysm due to recurrent abdominal pain. Ultrasonography revealed an abdominal mass. The patient had a history of hypertension, diabetes, and dyslipidemia. Laboratory results showed normal hemoglobin (14.3 Â g/dL) and mildly reduced eGFR (Ureum 37.3 mg/dL, Creatinine 1.19 mg/dL, eGFR 67 ml/min/1.73 m ^2^). CT-scan Angiography (CTA) revealed a challenging infrarenal AAA anatomy with a very short neck (9 mm), a maximum aneurysm diameter of 70.6 mm, and a length of 127 mm ( Figure 4A).

Aortography confirmed challenging anatomy, including a short neck, neck angulation, and a right common iliac aneurysm ( Figure 4B-D). After deploying the main body, coils of 6 × 6.5 mm (2 coils), 5 × 5.5 mm (2 coils), and 4 × 3.7 (1 coil) were used to occlude the right internal iliac artery ( Figure 4E-F). Ballooning was performed on both limbs; however, wire twisting in the contralateral limb required additional wiring through brachial access ( Figure 4G-J). The final aortography evaluation showed a good stent position and minimal type 1 B endoleak. After additional ballooning, final aortography showed no endoleaks ( Figure 4K). Total contrast was 230 cc, fluoroscopy time 1 hour 23 minutes, and CT showed no endoleaks.

The patient was discharged without any complaints after five days. Follow-up CTA 3 months after the procedure showed an excellent stent position at the infrarenal aorta until the left and right common iliac arteries without endoleaks ( Figure 4L).

### Case 4

**Figure 5.**
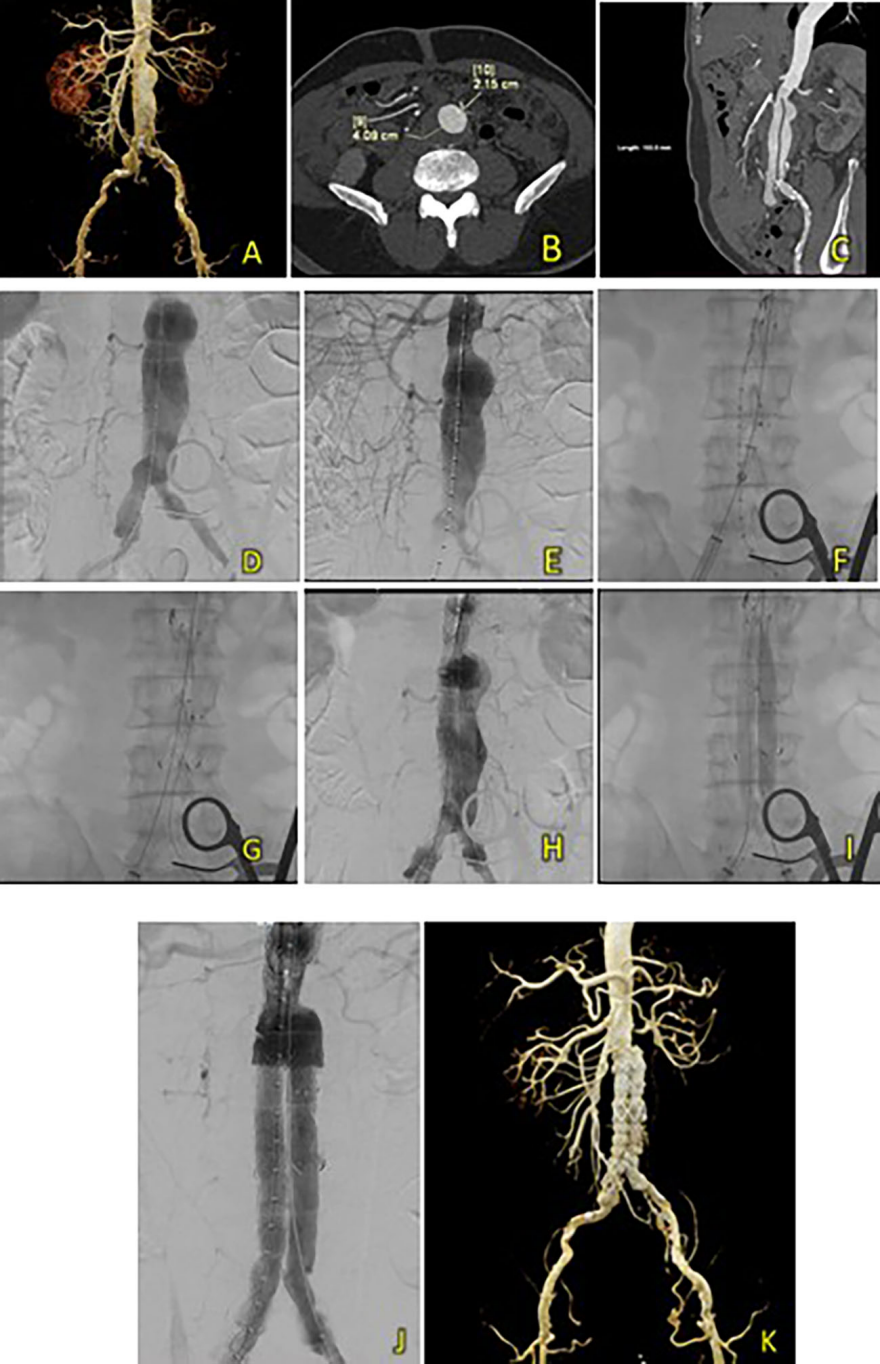
T&K B-EVAR on Patient 4. (A) Pre-procedural CT scan. (B) Axial CTA. (C) Sagittal CTA. (D-I) T&K B-EVAR step-by-step. (J) Post-procedure aortography with good stent apposition and no endoleaks. (K) Post-procedural CT scan evaluation had good results with no signs of endoleaks.

A 71-year-old man was admitted for EVAR due to a pulsing sensation in the stomach. The patient had a history of undergoing a CABG procedure approximately 11 years ago and had a history of hypertension. Laboratory results revealed a normal hemoglobin level (13.6 g/dL) and slightly decreased eGFR (Ureum 38.7 mg/dL, Creatinine 1.41 mg/dL, and eGFR 53 ml/min/1.73 m ^2^). CT-Scan Angiography (CTA) was performed. It showed an AAA with thrombus from the infrarenal region after the bilateral renal arteries to the aortic bifurcation around 103.55 mm in length with a maximum sac diameter of 40.9 mm, and right common iliac artery aneurysm with thrombus, as well as intermittent subtotal thrombus with irregularity in the bilateral internal iliac artery ( Figure 5A-C). The T&K B-EVAR procedure was performed.

After femoral artery access, a SEAL NOVUS Body Stent Graft was deployed through contralateral access, followed by limb extensions through both ipsilateral and contralateral access ( Figure 5D-I). Aortography confirmed good stent positioning with no endoleaks ( Figure 5J). A total of 90 mL of Ultravist 370 contrast was used, with a dose area product (DAP) of 51.722 Gy.cm ^2^. The fluoroscopy time was 17 minutes and 9 seconds.

After four days, the patient was discharged without any complaints. A follow-up CTA 1 month after the procedure revealed promising results: a well-expanded and apposed graft stent without an endoleak ( Figure 5K).

### Case 5

**Figure 6.**
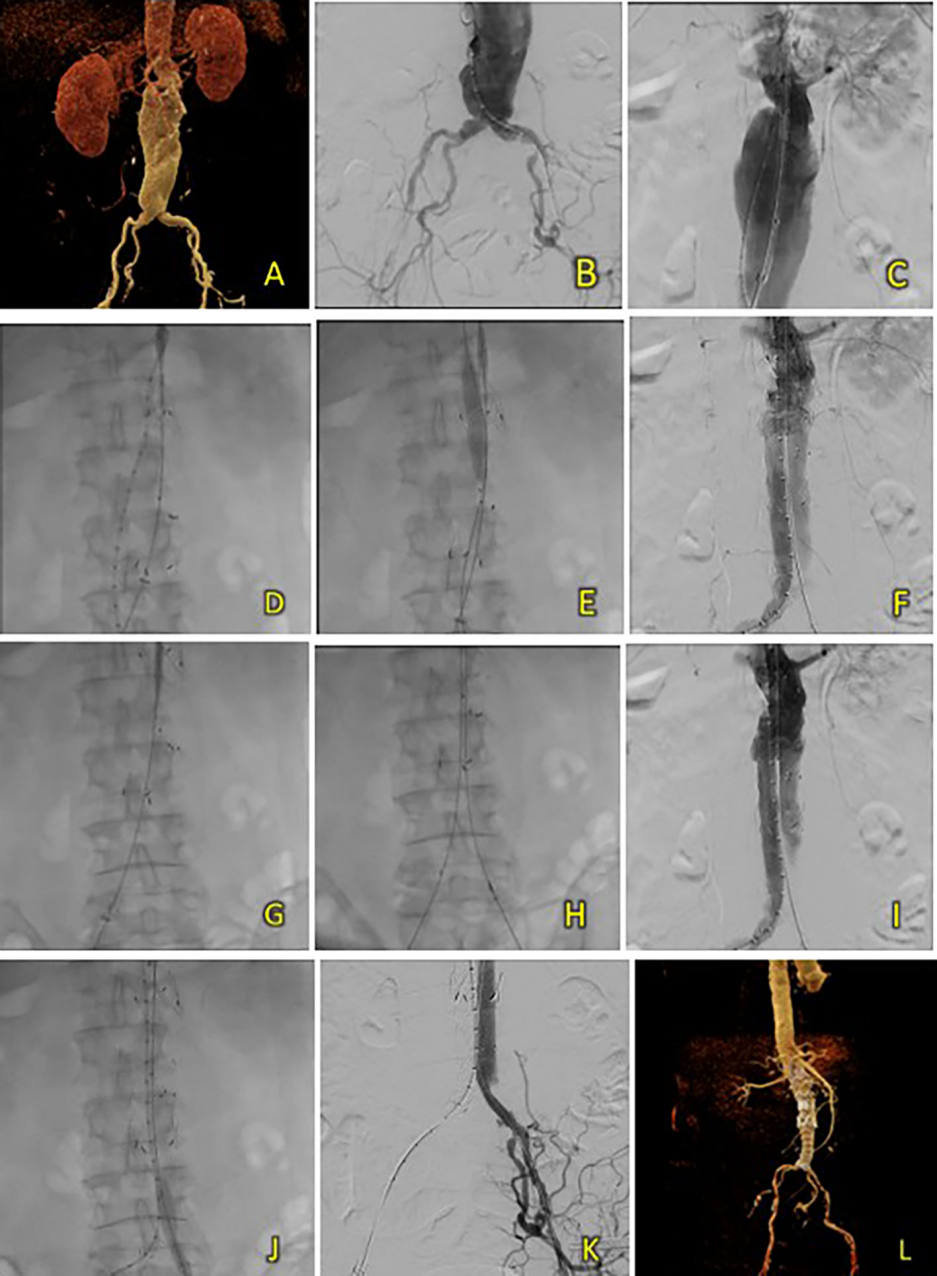
T&K B-EVAR on Patient 5. (A) Pre-procedure CT scan. (B-C) Procedural diagnostic angiography. (D-E) Deployment of main body stent. (F) Angiographic evaluation revealing the expansion of graft stent. (G-H) deployment and ballooning of extensions. (I) Angiographic evaluation with reduced flow through the left extension limb. (J) Ballooning of limb extension. (K) After the angiography evaluation after additional limb extension deployment, the results showed good flow and no endoleak. (L) Post-procedure CTA was showing promising results.

A 67-year-old man was admitted to our hospital with AAA and a history of hypertension and dyslipidemia. Laboratory results showed normal hemoglobin level (15.3 g/dL) and signs of renal insufficiency (Ureum 56.2 mg/dL, Creatinine 1.85 mg/dL, and eGFR 40 ml/min/1.73 m ^2^). A CT-Scan angiography (CTA) revealed a challenging AAA morphology with a conical-shaped neck, thrombus throughout the whole aneurysm from the infrarenal region to the aortic bifurcation around 113.7 mm in length, a maximum sac diameter of 58 mm, a very short common iliac artery (15.4 mm), and a small access diameter ( Figure 6A).

A T&K B-EVAR procedure was performed with a sheathless approach due to the small right iliac artery. After deploying the SEAL NOVUS Body Stent Graft and limb extensions ( Figure 6B-E), aortography revealed under-expansion near the left iliac artery, corrected by balloon inflation ( Figure 6F-J). An additional stent was placed to ensure full coverage. Final aortography showed good stent positioning, no endoleak, and adequate flow in the left iliac artery ( Figure 6K). Approximately 90 mL of Ultravist 370 contrast was used, with a DAP of 92.4 Gy.cm ^2^. Procedure time was 1 hour 32 minutes, and fluoroscopy time was 34 minutes 9 seconds.

The patient was discharged without any complaints after six days. A follow-up CTA 4 months after the procedure revealed promising results, a well-expanded and apposed graft stent without any endoleak ( Figure 6L).

### Case 6

**Figure 7.**
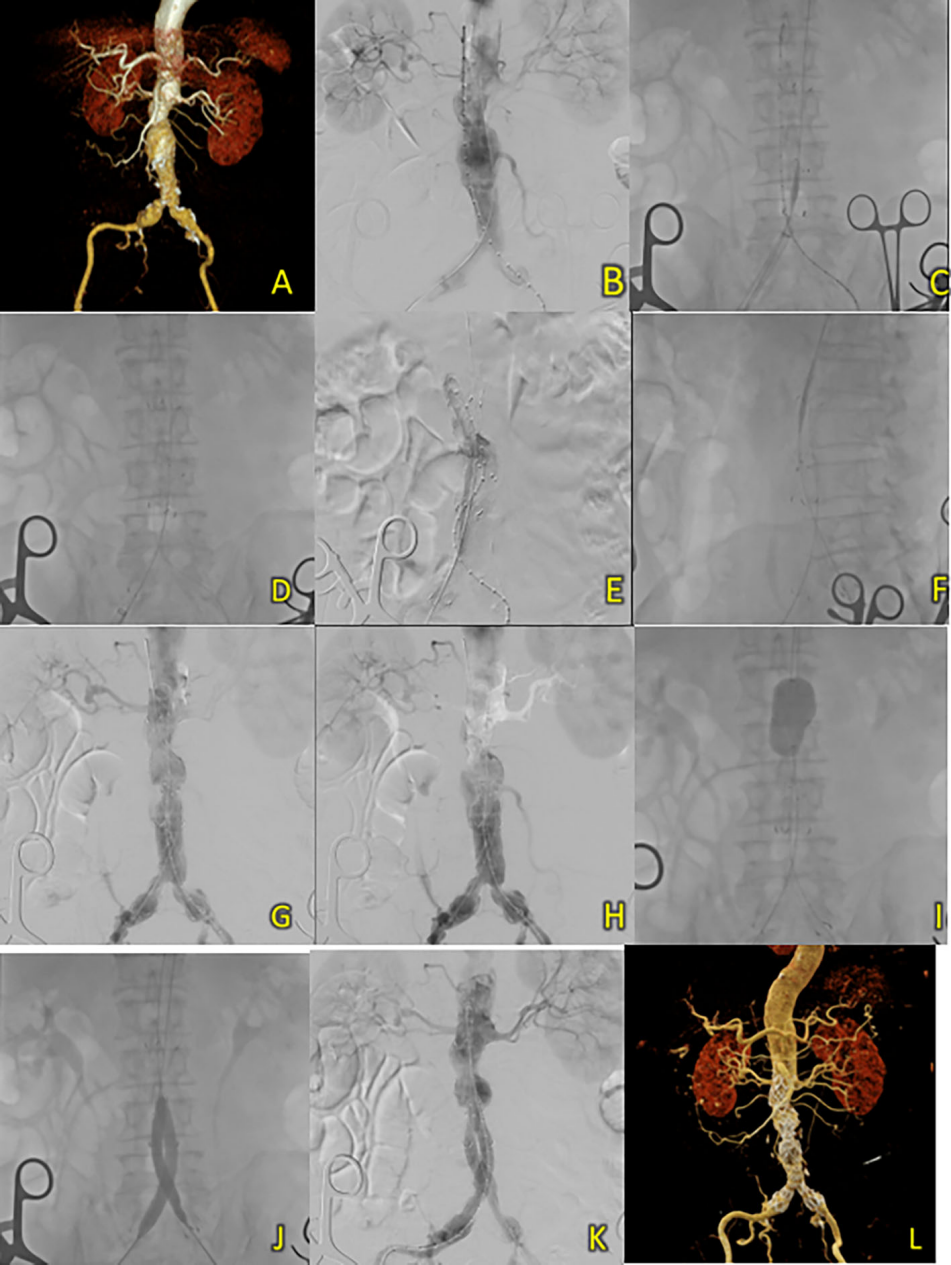
T&K B-EVAR on Patient 6. (A) Pre-procedure CTA. (B) Angiography reveals an infrarenal fusiform aneurysm. (C-F) T&K B-EVAR step-by-step. (G-H) Underexpansion of graft stent. (I-J) Balloon dilatation of main body graft and limb extensions. (K) Evaluation angiography reveals good graft placement and no endoleak. (L) Post-procedure CTA shows good results.

A 76-year-old man was admitted with a burning sensation in the chest and epigastric areas. He was suspected to have AAA and had a history of hypertension. Laboratory results showed a normal hemoglobin level (13.4 g/dL) and signs of chronic kidney disease (Ureum 26.1 mg/dL, Creatinine 1.83 mg/dL, and eGFR 38 ml/min/1.73 m ^2^). CT-Scan Angiography (CTA) was then performed, and it showed an AAA from the infrarenal region to the aortic bifurcation around 114.4 mm in length and a maximum sac diameter of 45.1 mm, and dilatation of the right common iliac artery with a diameter of 21.1 mm ( Figure 7A).

A T&K B-EVAR procedure was performed with access through the right and left femoral arteries. After deploying the SEAL NOVUS Body and limb Stent Grafts ( Figure 7B-F), aortography revealed under-expansion, especially in the main body and right iliac artery area ( Figure 7G-H). This was corrected with multiple balloon dilations at the proximal, main body, and iliac stents ( Figure 7I-J). Final aortography showed good stent positioning, no endoleak, and improved flow in both iliac arteries ( Figure 7K). A total of 180 mL of Metacosfar 320 contrast was used, with a DAP of 17.140 Gy.cm ^2^. Fluoroscopy time was 34 minutes 38 seconds.

After six days, the patient was discharged without any complaints. Follow-up CTA approximately 2 weeks after the procedure revealed minimal contrast leakage at the 4th and 5th lumbar vertebrae and a penetrating atherosclerotic ulcer (PAU) in the aortic isthmus ( Figure 7L).

## Discussion

EVAR has evolved rapidly over the past decade, leading to many advances in configuration and design. Cannulation of the contralateral gate remains one of the most challenging and time-consuming procedures in EVAR deployment, increasing procedural time, radiation exposure, and material costs resulting from any potential bailout strategy. ^11^^,^^12^ Even with experienced operators, this rate-limiting step can still be challenging as it requires the most wiring and catheter skills. This is especially relevant as the number of patients with complex aortoiliac anatomy who undergo EVAR continues to increase. Several anatomic features have been reported to prolong the time spent for CGC, namely maximal aneurysm diameter, active thrombus-free lumen, iliac tortuosity, and aortic bifurcation angulation. ^10^ Several experiments have shown a correlation between aortic neck angulation and prolonged CGC. Despite device improvements, it still depends on the operator’s skills and technical expertise.

The various cases presented highlight the potential universal applicability of the T&K B-EVAR device and method. Of the six reported cases, all had successful outcomes despite the difficult anatomy in some cases. Only one patient in our cohort required an additional adjunctive procedure using a snaring technique from the right brachial access to untwist the contralateral femoral wire. None of the patients experienced serious complications or were discharged with an acceptable LOS. In our case series, difficult cases were reported to have a longer fluoroscopy time and an overall longer procedure time. This finding was consistent with the current literature, which states that a more complex aortoiliac anatomy is associated with an increased procedure time.

Compared to normal bifurcated EVAR, traditional bifurcated EVAR is influenced by many factors, all of which increase contralateral gate cannulation time. Free-hanging bifurcation is susceptible to severe aneurysm neck angulation or highly splayed iliac arteries, which may cause narrowing or bending of gates and require additional adjunctive approaches. T&K B-EVAR offers a sophisticated and simplified approach in which the main body graft fabric extends to encapsulate the bifurcation. In theory, this special design enables easier cannulation, as cannulation is performed on the main body with a larger diameter. This design also means that the positioning strategies normally needed in traditional bifurcated EVAR are no longer required. Furthermore, the graft design offers extra structural support and ballooning of the ipsilateral gate enables easier wiring into the contralateral gate. The extra advantage offered by the device design can enable faster procedure time, reduce fluoroscopy time, and potentially reduce contrast usage and its associated complications, such as contrast-induced nephropathy. The potential reduction in cannulation difficulty also reduces the need for bail-out procedures, which adds to the procedure time and cost. Finally, this device was designed to be universally applicable and can be used in patients with various anatomical features. Therefore, the author posits that the B-EVAR device is essential due to its significant advantages over traditional bifurcated EVAR.

## Conclusion

T&K B-EVAR aims to simplify the endovascular AAA repair. The device design enables easier cannulation of the contralateral limb, thereby reducing the procedure time, radiation exposure, and the risk of endoleaks. This technique has been proven to be safe in six patients, reproducible, and potentially universally applicable. However, further research with a larger sample size is needed to validate these results.

### Ethics and consent

Written informed consent was obtained from the patient for the publication of the case series and accompanying images. We have obtained ethical approval to conduct this study from the National Cardiovascular Center Harapan Kita Hospital.

## Data Availability

All data underlying the results are available as part of the article and no additional source data are required. ^13^ Figshare. CARE checklist for ‘A Revolutionary Device for Endovascular Aortic Repair of Abdominal Aortic Aneurysm: A Pilot Study’. DOI: https://doi.org/10.6084/m9.figshare.23925234.v2 ^13^ Data are available under the terms of the Creative Commons Zero “No rights reserved’ data waiver (CC0 1.0 Public domain dedication).

## References

[1] Keisler B Carter C : *Abdominal aortic aneurysm.* American Family Physician;2015, April 15. 25884861

[2] Song P He Y Adeloye D : The global and regional prevalence of abdominal aortic aneurysms: A systematic review and Modeling Analysis. *Ann. Surg.* 2022;277(6):912–919. 10.1097/SLA.0000000000005716 36177847 PMC10174099

[3] Chan WK Yong E Hong Q : Systematic Review and meta-analysis of the prevalence of abdominal aortic aneurysm in Asian populations. *J. Vasc. Surg.* 2021;73(3):1069–1074.e1. 10.1016/j.jvs.2020.08.140 32987145

[4] Golledge J Thanigaimani S Powell JT : Pathogenesis and management of abdominal aortic aneurysm. *Eur. Heart J.* 2023;44(29):2682–2697. 10.1093/eurheartj/ehad386 37387260 PMC10393073

[5] Ullery BW Hallett RL Fleischmann D : Epidemiology and contemporary management of abdominal aortic aneurysms. *Abdom. Radiol.* 2018;43(5):1032–1043. 10.1007/s00261-017-1450-7 29313113

[6] Tenorio ER Dias-Neto MF Lima GB : Endovascular repair for thoracoabdominal aortic aneurysms: Current status and future challenges. *ASVIDE.* 2021;8:326–326. 10.21037/asvide.2021.326 PMC8640886 34926178

[7] Calligaro KD Toursarkissian B Clagett GP : Guidelines for hospital privileges in vascular and endovascular surgery: Recommendations of the Society for Vascular Surgery. *J. Vasc. Surg.* 2008;47(1):1–5. 10.1016/j.jvs.2007.10.003 18060729

[8] Rehman ZU Choksy S Howard A : Comparison of patient radiation dose and contrast use during EVAR in a dedicated hybrid vascular or and mobile imaging. *Ann. Vasc. Surg.* 2019;61:278–283. 10.1016/j.avsg.2019.04.019 31336160

[9] Koussayer S Abuduruk A Alkhezzi A : Anatomy-based strategies for contralateral gate cannulation during evar. *Ann. Vasc. Surg.* 2022;2(2):100088. 10.1016/j.avsurg.2022.100088

[10] Wolf YG Tillich M Lee WA : Impact of aortoiliac tortuosity on endovascular repair of abdominal aortic aneurysms: evaluation of 3D computer-based assessment. *J. Vasc. Surg.* 2001;34(4):594–599. 10.1067/mva.2001.118586 11668310

[11] Pakeliani D Lachat M Blohmé L : Improved technique for sheath supported contralateral limb gate cannulation in endovascular abdominal aortic aneurysm repair. *Vasa.* 2019;49:39–42. 10.1024/0301-1526/a000820 31549917

[12] Titus JM Cragg A Alden P : A prospective randomized comparison of contralateral snare versus retrograde gate cannulation in endovascular aneurysm repair. *J. Vasc. Surg.* 2017;66(2):387–391. 10.1016/j.jvs.2017.01.038 28433339

[13] Taofan : Daftar pasien T&K B-EVAR.[Data set]. National Cardiovascular Center Harapan Kita Academic Hospital;2023.

