# Supplementary material for: A Revolutionary Device for Endovascular Aortic Repair of Abdominal Aortic Aneurysm: A Pilot Study
Source: F1000Res. 2024 Nov 6;13:892. Originally published 2024 Aug 6. [Version 2] doi: 10.12688/f1000research.152060.2 (PMC11584456; doi:10.12688/f1000research.152060.2)
Supplement: Peer review history

# Reviewer report: Reviewer response for version 2

**DOI:** 10.5256/f1000research.174220.r338327

**Published:** 2024-11-22

**Authors:** Elena Giacomelli, Walter Dorigo

**Affiliations:** 1 Azienda Ospedaliero-Universitaria Careggi, Florence, Italy

The answers fulfilled the questions we provided.

Are enough details provided of any physical examination and diagnostic tests, treatment given and outcomes?

Yes

Is the background of the cases’ history and progression described in sufficient detail?

Yes

Is sufficient discussion included of the importance of the findings and their relevance to future understanding of disease processes, diagnosis or treatment?

Partly

Is the conclusion balanced and justified on the basis of the findings?

Partly

Reviewer Expertise:

vascular surgery

We confirm that we have read this submission and believe that we have an appropriate level of expertise to confirm that it is of an acceptable scientific standard.

# Reviewer report: Reviewer response for version 1

**DOI:** 10.5256/f1000research.166766.r318472

**Published:** 2024-09-16

**Authors:** Elena Giacomelli, Walter Dorigo

**Affiliations:** 1 Azienda Ospedaliero-Universitaria Careggi, Florence, Italy

The authors describe six cases of AAA treated with a new endograft made of a main body stent graft and two limb extensions.

 The paper is well written; probably the case presentation is redundant and should be shortened.

 The most important limit of the study is the absence of the definition of the anatomic criteria for inclusion and exclusion. The reasons for using such a technique in the selected cases should be fully described.

 Moreover it is not clear from the text whether the septum inside the main body extends all over the graft or it is interrupted after a certain numbers of centimeters. This is crucial to understand if adjunctive procedures from the contralateral side are possible or not.

 Finally the authors should state the reasons why they consider this kind of device really necessary in this era of very complex and advanced endografts allowing the treatment of complex lesions and anatomies. In consideration of this, the claim of the title should be tempered.

Are enough details provided of any physical examination and diagnostic tests, treatment given and outcomes?

Yes

Is the background of the cases’ history and progression described in sufficient detail?

Yes

Is sufficient discussion included of the importance of the findings and their relevance to future understanding of disease processes, diagnosis or treatment?

Partly

Is the conclusion balanced and justified on the basis of the findings?

Partly

Reviewer Expertise:

vascular surgery

We confirm that we have read this submission and believe that we have an appropriate level of expertise to confirm that it is of an acceptable scientific standard, however we have significant reservations, as outlined above.

# Response

**Published:** 2024-10-29

**Authors:** Taofan Taofan

**Affiliations:** National Cardiovascular Center Harapan Kita, Indonesia

**Dear Reviewer,**

 Thank you for your thoughtful and detailed review of our manuscript. We greatly appreciate your constructive feedback, which has provided valuable insights for improving the clarity and scientific rigor of our study.

**Case Presentation Streamlining**

 We agree with your assessment that certain case details may have been repetitive. In response, we will streamline the case presentations, focusing on the most relevant clinical outcomes and procedural specifics. This will ensure the inclusion of critical information while eliminating unnecessary repetition, enhancing the overall clarity of the manuscript.

**Indications and Exclusion Criteria**

 This device has been developed primarily to enhance the technical feasibility of EVAR, addressing several limitations observed in current bifurcated designs. We believe it offers a significant improvement in anatomical fit and procedural simplicity, making it suitable for patients with anatomies that may present challenges to conventional devices. Here are the key features that make B-EVAR more feasible:

1. **Long Trunk Design and Increased Flexibility:**
  The B-EVAR device features a long trunk type design, which provides greater flexibility compared to current bifurcated designs with shorter main body lengths. This added flexibility enables the device to adapt better to the aorta and iliac arteries, particularly in patients with tortuous anatomy, reducing the risk of device malposition or kinking during deployment.
2. **Absence of Bifurcation and Related Complications:**
  The B-EVAR device eliminates the bifurcation point, which is a common source of complications in traditional EVAR. By avoiding the modular connection at the bifurcation, this design reduces the risk of modular leaks and bifurcation kinks, both of which are significant causes of post-procedure failure in standard bifurcated systems.
3. **Large Distal Skirt for Easier Contralateral Limb Selection:**
  One of the most critical innovations in the B-EVAR design is the large distal skirt. This feature significantly facilitates contralateral limb selection, a step that can often be technically challenging in bifurcated systems. With the B-EVAR device, the wide skirt allows for easier access and cannulation of the contralateral limb, improving procedural efficiency and reducing overall procedure time, radiation exposure, and contrast usage.

**Septum Design Clarification**

 Your observation regarding the septum inside the main body of the stent graft is highly appreciated. We have revised the manuscript to specify that the septum extends only into the lower middle portion of the main body and is interrupted after a certain distance. The septum does not extend to the distal portion of the main body. This design allows for adjunctive procedures from the contralateral side when necessary, ensuring procedural flexibility.

**Necessity of the B-EVAR Device and Title Modification**

 You also inquired about the necessity of this device in an era of highly complex and advanced endografts. While we acknowledge that many sophisticated endografts exist, the B-EVAR device simplifies the often-challenging step of contralateral gate cannulation in complex anatomies. The device’s novel design helps reduce procedure time, radiation exposure, and contrast usage, making it a valuable option in cases where anatomical complexity could prolong the procedure. In light of your suggestion, we have tempered the claim in the title to reflect these considerations more accurately. We believe this approach will pave the way for future EVAR devices that prioritize bidirectional methods over traditional bifurcated systems.

**Conclusion**

 We hope this clarification provides a better understanding of the indications for using the B-EVAR device. Its flexibility, avoidance of bifurcation-related complications, and ease of contralateral limb selection make it a highly feasible and advantageous alternative to traditional bifurcated EVAR devices.

 We look forward to your further comments and suggestions, and we thank you again for the opportunity to refine our manuscript.

 Best regards,

 Taofan

 Department of Cardiology and Vascular Medicine

 Universitas Indonesia

# Reviewer report: Reviewer response for version 1

**DOI:** 10.5256/f1000research.166766.r314925

**Published:** 2024-09-13

**Authors:** Muhamad Taufik Ismail

**Affiliations:** 1 Sardjito Central General Hospital/Faculty of Medicine, Public Health, and Nursing, Gadjah Mada University,, Yogyakarta, Indonesia

Congratulations on the development of the T&K B-EVAR device, a significant advancement in endovascular aortic repair that enhances the process of contralateral gate cannulation. I have a few suggestions to further strengthen your manuscript:

1. **Provide a Detailed Comparative Analysis:** Include a more comprehensive comparison between the T&K B-EVAR device and existing devices and techniques. This would help to clearly delineate the unique advantages and improvements offered by your device.
2. **Discuss the Limitations:** Explicitly outline the limitations observed in your cases. A transparent discussion of these limitations will provide a more balanced perspective and help readers assess the broader applicability of your findings.

Are enough details provided of any physical examination and diagnostic tests, treatment given and outcomes?

Yes

Is the background of the cases’ history and progression described in sufficient detail?

Yes

Is sufficient discussion included of the importance of the findings and their relevance to future understanding of disease processes, diagnosis or treatment?

Yes

Is the conclusion balanced and justified on the basis of the findings?

Yes

Reviewer Expertise:

vascular medicine, peripheral intervention, angiologist, cardiovascular intervention

I confirm that I have read this submission and believe that I have an appropriate level of expertise to confirm that it is of an acceptable scientific standard.

# Response

**Published:** 2024-09-14

**Authors:** Taofan Taofan

**Affiliations:** National Cardiovascular Center Harapan Kita, Indonesia

**Dear dr. Taofik,**

 Thank you for taking the time to review my article. I greatly appreciate your thoughtful comments and constructive suggestions. Your insights have been invaluable in helping me improve the quality and clarity of the work. I have carefully considered all your feedback and have made the necessary revisions to address the points raised.

 Once again, thank you for your thorough review and your contribution to enhancing this manuscript.

 Sincerely,

 Taofan,

 Department of Cardiology and Vascular Medicine Universitas Indonesia
